# Influence of Rice Flour, Glutinous Rice Flour, and Tapioca Starch on the Functional Properties and Quality of an Emulsion-Type Cooked Sausage

**DOI:** 10.3390/foods9010009

**Published:** 2019-12-20

**Authors:** Jailson Pereira, Hongyan Hu, Lujuan Xing, Wangang Zhang, Guanghong Zhou

**Affiliations:** Key Laboratory of Meat Processing and Quality Control, Ministry of Education China, Jiangsu Collaborative Innovation Center of Meat Production and Processing, Quality and Safety Control, College of Food Science and Technology, Nanjing Agricultural University, Nanjing 210095, China

**Keywords:** cereal products, starch, sausage, meat emulsion, microstructure

## Abstract

This study aimed to investigate the impact of the addition of rice flour (RF) and glutinous rice flour (GRF) in comparison with tapioca starch (TS) on the emulsion stability, water states, protein secondary structure, and microstructure of an emulsion-type cooked sausage. Their incorporation significantly increased the cooking yield and moisture retention of cooked sausages (*p* < 0.05). RF and GRF significantly decreased the fat loss and total fluid release of the cooked sausage compared to control (*p* < 0.05). However, RF and GRF functional effects on these parameters remained lower compared to TS (*p* < 0.05). Among these functional ingredients, TS had a significantly higher emulsion stability and cooking yield and generated a firmer and more uniform gel network structure. The transverse relaxation time T2 results revealed four categories of water population (T_2b1_, T_2b2,_ T_21,_ and T_22_) with particular mobility. TS immobilized a greater proportion of water molecules within the myofibrils (T_21_ population). All three ingredients increased the emulsion stability of the emulsion-type cooked sausages by decreasing the fat globule mobility and binding more water molecules. Raman spectra (400–3600 cm^−1^) showed that the incorporation of RF, GRF, or TS did not affect the β-sheet and α-helix protein structure. However, TS presented significantly higher contents of the random coil structure. These findings provide a good insight into the effects of RF and GRF as functional ingredients to manufacture emulsified meat products with good quality and improved nutritional values.

## 1. Introduction

Interactions between water, muscle protein, and fat globules are the primary factor responsible for the desired quality and structural organization of a large number of emulsified meat products. However, many plant-based ingredients, including starch, fibers, and proteins, are efficiently used to stimulate the formation of protein gel during the cooking process, which subsequently improves the quality and nutritional value of cooked emulsified meat products [1,2,3].

Starch is a carbohydrate polymer derived from plant-based sources that exert functional roles in processed food, including meat products, by acting as a thickening, stabilizing, and binding agent as well as a filling agent to lower the cost of the formulation [2,4]. The functionality of different types of starches depends on many factors, including their source, granular structure, amylose and amylopectin content, and branch chain length distribution.

Generally, these functional ingredients are polysaccharides formed by different ratios of amylose, which is responsible for the gel strength, and amylopectin, which is responsible for the viscoelastic properties of a starch gel [2,5]. This functional property is related to the capability of the starch to gelatinize during the thermal process, which consequently helps to bind and retain large amounts of water in the system [6,7]. The interactions of these ingredients with muscle protein could directly influence structural and physicochemical properties by binding water, stabilizing fat, producing desirable textural properties, and achieving cohesion. An improvement in the texture and organoleptic quality of emulsified meat products was found by using hydrocolloids, such as cereal flour and starches, in emulsified meat products [8,9].

Tapioca starch (TS) is well used in the processing of meat products because of its surface sheen, smooth texture, and neutral taste [3,9]. Cereal products derived from milled cereal grains, such as rice flour (RF) and glutinous rice flour (GRF), mainly composed of starch (70%–80%), were also used in the meat emulsion to retain water, bind meat particles, and enhance texture as well as help in the gel formation and structure of final product [8,10]. Glutinous rice flour has a soft and sticky nature that could favor the stability of the emulsified meat product [6]. This cereal flour could provide good nutritional value and allow the production of gluten-free meat products with healthier characteristics. However, it is crucial to provide deep insight into the functionality of the meat emulsion system induced by plant-based starch molecules and meat protein, which are essential components in the development of emulsified meat products. Thus, the current study was proposed to explore the additional effects of rice flour and glutinous rice flour in comparison with tapioca starch on the cooking yield, water state, protein secondary structure, and microstructure of an emulsion-type cooked sausage.

## 2. Materials and Methods

Pork leg lean meat (71.58% moisture, 22.40% protein, 6.02% fat, pH 5.7) and back-fat were purchased from Yurun Food Co., Ltd. (Nanjing, China). RF and GRF were obtained from Wenfeng Refined Oil and Rice Ltd., Jiangsu, China. TS was obtained from a commercial supplier (Heely Food, Shanghai, China). Other ingredients (salt, white pepper, sodium tripolyphosphate) were provided by the pilot lab of the National Center of Meat Research, Nanjing Agricultural University, China. RF comprised 10.11% moisture, 7.46% protein, 1.57% fat, 1.02% ash, and 79.84% carbohydrates. GRF comprised 9.64% protein, 12.42% moisture, 2.15% fat, 0.87% ash, and 74.92% carbohydrates. TS comprised 11.26% moisture, 0.32% ash, and 88.42% carbohydrates [10].

### 2.1. Preparation of an Emulsion-Type Cooked Sausage

Pork leg lean meat, purchased within 24 h post-mortem, and back-fat were chopped separately by a chopper (TC 12E, Sirmam, Venezia, Italy) at low speed and stored at −20 °C until sausage processing. Three replications of emulsion-type sausages per formulation (Table 1) were manufactured on three different days according to the procedure described by Pereira et al. [7] with slight modifications. TS was used as a positive control because it is commonly used as an ingredient to improve the functional properties of emulsified meat products, while sausage formulated without plant-based starch was used as a negative control.

The previously chopped lean meat was transferred to a food processor (BZBJ-15, Expro Stainless Steel Mechanical & Engineering Co., Ltd., Hangzhou, China) and chopped for 3 min with salt and half of the ice and water. Then, pork back-fat, the remaining ice and water, RF, GRF, or TS treatments, and other seasonings were separately added to the batter and chopped for a further 3 min at high speed. The temperature of the raw batter was kept below 10 °C during the 6 min of the mixing process. The raw batter of each formulation was stuffed into casings of 25 mm diameter (Baan Thai Food Company, China). Cooked sausages were obtained after heating the sample in a water bath for 30 min at 80 °C. Approximately 400 g of raw batter of sausage were transferred to a laminated film (nylon/polyethylene) bag, vacuum-packaged, and stored at 4 °C until further analysis.

### 2.2. Proximate Composition and pH Measurement

Proximate composition values of cooked sausages, including protein, moisture, and fat content, were measured according to the Association of Official Analytical Chemists [10] methods 992.15, 950.46, 985.15, and 920.153, respectively. The pH was measured after homogenizing 10 g of sausage samples with 40 mL of distilled water collected from the Milli-Q apparatus (Millipore SAS, Molsheim, France).

### 2.3. Cooking Yield and Moisture Retention

The cooking yield and moisture retention of sausage samples were analyzed according to methods referenced by Gao et al. [6] with minor modification. Samples (40 g) were placed into a weighed centrifuged tube and cooked at 80 °C in a water bath for 30 min. Then, the fluid released was completely removed after cooling the samples at room temperature. The weights of the cooked samples were taken, and the results were calculated according to the following equations:

Cooking yield% = Weight of cooked samples/Weight of raw sample × 100%,

Moisture retention = Cooking yield% × Moisture of cooked samples% /100.

### 2.4. Emulsion Stability

Emulsion stability of the sausage was measured after heating the sample in a water bath following the protocol used by Hu et al. [11] with slight modification. Specifically, 50 g of raw batter were transferred to an 80 mL centrifuge tube and centrifuged at 1000× *g* for 3 min at 4 °C to remove air bubbles (Allegra 64R, Fisher Scientific, Pittsburgh, PA, USA). Each tube was heated in a water bath for 30 min until it reached 80 °C. Then, it was cooled to room temperature and the expressible fluid that was released was transferred to pre-weighed crucibles. The crucible with fluid was dried at 105 °C for 16 h to measure the volume of total fluid release (TFR) and fat loss (FL) throughout the following equation:

TFR% = TFR/Initial sample weight × 100

FL% = (Crucible + Dried supernatant weight) − (Weight of empty crucible)/TFR × 100

### 2.5. Texture Profile Analysis

The texture profile analysis of the emulsion-type cooked sausage was performed in a texture analyzer XT Plus (Stable Micro Systems Ltd., Godalming Surrey, UK) with probe P50, following the procedures of Pereira et al. [7]. The values for the hardness, springiness, cohesiveness, and chewiness properties were determined. The mean values of six replications were recorded for each batch.

### 2.6. Water State Measurements

The measurements of water mobility and distribution in the sausage samples were performed using low-field nuclear magnetic resonance (LF NMR) instrument (PQ001, Niumag Corporation, Shanghai, China) according to the procedure described by Zheng et al. [12]. Approximately 2 g was cut (3 cm long and 1 cm in diameter) from the interior of each cooked sausage and transferred to a cylindrical glass tube. The samples were inserted into the NMR probe after calibrating the instrument. Analyses were performed at 32 °C with a resonance frequency of 22.4 MHz. The Carr–Purcell–Meiboom–Gill sequence was applied to measure the proton transverse T2 relaxation time. The T2 relaxation time was measured with a τ-value (time between 90° pulses and 180° pulses) of 100 μs. T2 relaxometry data from 5000 echoes were obtained as 16 scan repetitions. Each sample was measured four times and analyzed using a multi-exponential model (Multi Exponential. Inv. Analysis, Niumag Corporation, Shanghai, China).

### 2.7. Raman Spectroscopy Measurements

The secondary structure of sausage samples was analyzed by exposing the sample to the Raman apparatus, as described in the protocol of Xue et al. [13]. Sample spectra were recorded in the 400 cm^−1^ to 3600 cm^−1^ resolution range. The spectrum of each sample was obtained by exposing the raw sample to the following conditions: 3 scans, 30 s of exposure time, 2 cm^−1^ resolution, 120 cm^−1^/min of sampling speed, and data collection every 1 cm^−1^. Labspec software v.5 (Horiba/Jobin Yvon, Longjumeau, France) was used to smooth, baseline correct, and normalize the spectra against the phenylalanine band 1003 cm^−1^ according to the explanation of Herrero et al. [14]. The modifications in the protein secondary structure of an emulsion-type sausage were expressed as the percentage of α-helix, β-sheet, β-turn, and random coil structures.

### 2.8. Microstructure Evaluation

The structural conformations of sausage samples were analyzed to evaluate their structure, according to the protocol described by Hu et al. [11]. Cubic samples (3 × 3 × 3 mm^3^) cut from the internal part of the sausages were fixed with 3% of glutaraldehyde in 0.1 mol/L phosphate buffer (pH 7.3). The samples were post-fixed with osmium tetroxide (1%) prepared in 0.2 mol/L phosphate buffer (pH 7.3). Samples were washed and dehydrated in various concentrations of ethanol solutions. After that, the sample was dried, sputter-coated with gold/palladium (Denton 503 High-vacuum Evaporator, Moore Town, NJ, USA) and scanned using Hitachi S-3000 N microscopy (Hitachi High Technologies Corp., Tokyo, Japan) at 10 kV. Several micrographs were performed at 500× magnification to select the most representative ones.

### 2.9. Statistical Analysis

Statistical analysis of the recorded data was carried out using SPSS v.18.0 (SPSS Inc., Chicago, IL, USA). One-way ANOVA and Duncan’s multiple range tests were performed to determine the variance between the treatments. The graphs were generated using GraphPad PRISM software version 5 and Microsoft Excel 2010. The results were presented as the mean ± standard deviation, and statistical significance was expressed at a level of *p* < 0.05.

## 3. Results

### 3.1. Proximate Composition of Emulsion-Type Sausage

Table 2 shows the approximate composition of an emulsion-type sausage formulated with rice flour, gelatinous rice flour, or tapioca starch. The pH values among all cooked samples ranged from 6.57 to 6.70. Sausage samples formulated with cereal flour and starch ingredients significantly differed from the control regarding the pH values (*p* < 0.05). GRF showed a significantly higher protein content, followed by the control samples, compared to the addition of TS and RF (*p* < 0.05). This difference in protein content may be related to greater amounts of protein present in GRF. Emulsion-type sausage prepared with RF and GRF showed the highest moisture values compared to the control (*p* < 0.05), whereas no difference in the moisture content between cereal flours and TS samples was observed (*p* > 0.05). This suggests that the functional properties of the amylopectin present in the starch added to the meat batter could induce the absorption of more moisture during the gelatinization process. This statement is in agreement with Gao et al. [6], who explained that the moisture improvement and greater fat retention in pork patties were due to the high content of amylopectin present in the composition of GRF. Likewise, Pereira et al. [7] showed that the moisture content tended to be higher in emulsion-type meat products incorporated with starch-based ingredients compared to the control.

Incorporation of RF, GRF, or TS significantly reduced the percentage of fat content in the sausage samples compared to the control (*p* < 0.05). The addition of RF and GRF had a similar effect on the reduction of fat content compared to TS (*p* < 0.05). These ingredients could somehow fill the gaps left by the decrease in fat and provide more high-molecular carbohydrates to the emulsion-type sausage. The research findings of Ali et al. [8] also demonstrated a reduced fat content in cooked frankfurters prepared with rice flour.

### 3.2. Cooking Yield and Emulsion Stability

The results of cooking yield and thermal stability of the sausage formulated with RF, GRF, or TS are shown in Table 2. Incorporating RF, GRF, or TS significantly improved the cooking yield of emulsion-type sausage compared to the control, which was prepared without plant-based starch (*p* < 0.05). Similarly, Gao et al. [6] found that the addition of GRF and corn starch effectively improved the cooking yield of meat patties. Among treated sausage samples, RF and GRF had significantly higher cooking yield compared to the control (*p* < 0.05). However, the ability of RF and GRF to augment the cooking yield of the emulsion-type sausage was significantly lower compared to TS (*p* < 0.05). This fact suggests that pure starch (TS) could provide more benefits to meat emulsion compared to flour (RF, GRF), which contains a high content of starch. An increase in the cooking yield of pork meat patties and beef meat patties formulated with cereal flour was previously reported [6,8]. The use of plant-based starch components as non-meat ingredients could improve the water-binding ability of emulsified meat products by absorbing moisture during the gelatinization process [15]. Starch can induce the formation of a firm gel structure during the heating process by swelling the starch granules embedded in the protein matrix and consequently increasing the water-binding capability within the system [2,9]. As shown in Table 2, the incorporation of RF, GRF, or TS significantly improved the emulsion stability of the emulsion-type cooked sausages (*p* < 0.05). TS had substantially higher moisture retention and emulsion stability compared to RF, GRF and control (*p* < 0.05). The addition of TS allowed greater water binding in the cooked sausages due to higher starch gelatinization during the heating process. Our results corroborate the findings of Hughes et al. [9] who showed that tapioca starch (3%–6%) increased the emulsion stability of low-fat frankfurters by reducing fat globule mobility and binding more water molecules.

Similarly to TS, GRF and RF were useful to retain the fat particles during the heating process, which subsequently improved the emulsion stability of the final product. Other works have suggested that most filler agents or binders used as functional ingredients in meat emulsion increase the yield and improve the emulsion stability due to their functional properties that allow them to bind and retain water during the heating process [5,16]. Likewise, the study of Barbut [16] successfully decreased cooking loss by incorporating starch ingredients and improved the emulsion gel formation, resulting in the higher yield production of emulsion-type meat products. The fat loss in the sausage samples significantly decreased after the addition of RF, GRF, or TS compared to the control (*p* < 0.05). Similar effects in the fat loss were observed between GRF and TS, which suggests that the sticky nature of GRF plays some role in its fat binding properties. This functionality may be attributed to the binding ability of these ingredients and the lower amount of fat added to the formulation compared to control. In contrast, Barbut [16] suggested that the ability of starch to lower fat loss was highly associated with its viscosity properties. The starch swelling could favor its interactions with muscle protein within the emulsion, which subsequently prevents the migration or loss of fat from the emulsion-type cooked sausage.

### 3.3. Texture Profile Analysis

Texture profile analysis is considered to be an important aspect in determining sausage quality. According to the results displayed in Table 3, the incorporation of RF had significant effects in the emulsion-type cooked sausage compared to GRF, TS, and the control (*p* < 0.05). Similar hardness values were found between the control and GRF samples (*p* > 0.05), whereas both significantly differed from sausages formulated with RF and TS (*p* < 0.05). These results showed a similar trend compared to studies carried out by Yi et al. [17] and Gao et al. [6] who used 3% glutinous rice flour in meat patties. These authors found lower hardness values for meat patties containing GRF compared to the control and other treatments. Gao et al. [6] also suggested that the ability of GRF to lower the hardness values of sausage samples was associated with a high content of amylopectin.

Incorporation of TS significantly increased the hardness value compared to the control and samples containing RF and GRF (*p* < 0.05). The firmer texture of emulsion-type cooked sausage is correlated with the coagulation of proteins, starch gelatinization, and partial dehydration of the meat batter during the heating process. RF and GRF did not have a significant difference in springiness, cohesiveness, and chewiness values compared to the control (*p* > 0.05). This suggests that RF and GRF components, including starch and protein, similarly influenced the textural aspects of the cooked sausage but differed in hardness values to some extent. Incorporation of RF and GRF generated an emulsion-type sausage with less elastic structures, as revealed by the lower springiness value compared to TS (*p* < 0.05). TS had a greater ability to influence and strengthen the interaction between the components added to emulsified sausage in this study. Overall, the incorporation of TS had a better impact on the cooking properties and texture parameters of the emulsion-type cooked sausage.

### 3.4. Water State

**Figure 1:**
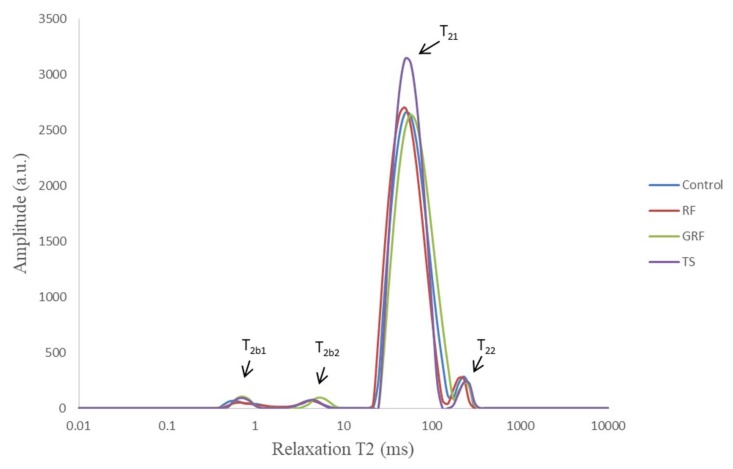
Low-field NMR T2 relaxation curves of an emulsion-type sausage formulated with rice flour (RF), glutinous rice flour (GRF), or tapioca starch (TS). T_2b1_ mean water associated with weak emulsion gel, T_2b2_ mean macromolecular-associated water, T_21_ mean entrapped water within intramyofibrillar, T_22_ mean free or extramyofibrillar water.

The water proton mobility and distribution, analyzed by the LF-NMR tool, reflect how the water and microstructural characteristics of the meat protein matrix are affected by their composition, type of ingredients added, and processing conditions, including heating or freezing processes. As shown in Figure 1, the transverse relaxation time (T2) curves of an emulsion-type sausage formulated with RF, GRF, or TS had a multi-exponential distribution with four distinct peaks (T_2b1_, T_2b2,_ T_21,_ and T_22_) of water populations that were approximately located between 0.5–1, 3–10, 40–90, and 200–400 ms respectively. The growth of these four different T2 relaxation time water fractions could be induced by the components surrounding the protein network and by the distance between the water proportions distributed in the emulsion systems. T_2b2_ represents water closely associated with macromolecular structures [18] and macromolecular protons in water-plasticized structures [19]. T_21_ is classified as entrapped or immobilized water molecules (82%–90%) within the myofibrils with relaxation times ranging from 10 to 100 ms [20]. T_22_ represents water located between fiber bundles or water molecules outside the highly compacted protein matrix with relaxation times ranging from 100 to 400 ms [18,21].

The first water component (T_2_b_1_) detected was associated with the lack of a strong and well-organized composite system gel structure, as suggested by García García et al. [22]. Other authors [23] have indicated that the shortest T2 relaxation time could be associated with water within the cell wall of the samples. The intramyofibrillar water proportion in the meat emulsion tends to decrease during the heating process, which generates more extramyofibrillar water and subsequently more drip loss. However, the use of plant-based starch as filler and binder ingredients could immobilize more water within the protein matrix from being expelled during gel-forming. The T_21_ component, located in well-organized and gelatinized structure, was enhanced by the presence of starch in RF, GRF, and TS compared to the control. TS immobilized more water compared with RF and GRF in the emulsion-type cooked sausages. This might be caused by the greater ability of TS to swell and absorb water molecules in the meat emulsion. The incorporation of starch could induce an initial high viscosity due to a consequent granular swelling, water imbibition, and possible solubility. Therefore, the slight changes in the water state that were observed may be due to a lower concentration of starch added during sausage manufacturing. The improvement in immobilized water by the added starch reflects the enhancement of moisture retention as well as better network structure in the final product.

**Figure 2:**
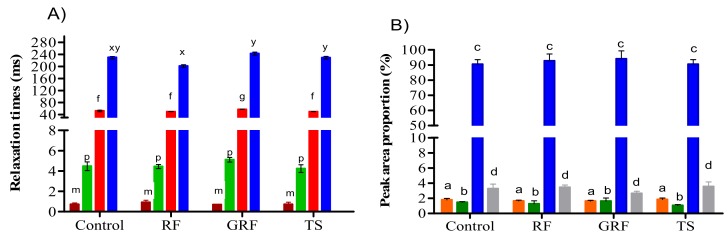
Relative values of T2 relaxation times (T_2b1_ ■, T_2b2_ ■, T_21_ ■, and T_22_ ■) and water proportions peak area (P_2b1_ ■, P_2b2_ ■, P_21_ ■, and P_22_ ■) of an emulsion-type sausage formulated with rice flour (RF), glutinous rice flour (GRF), or tapioca starch (TS). The significant difference in (**A**) T2 relaxation time was represented by letters (f, g, m, p, x, y) and (**B**) (a, b, c, d) for each water proportion peak area per treatment (*p* < 0.05). Treatments with the same letter showed no significant difference for each (**A**) T2 relaxation time and (**B**) water proportion, respectively (*p* > 0.05).

Low filed NMR T2 relaxation time measurements demonstrated that the addition of these ingredients could induce the transference of water molecules from a loosely bound to tightly bound water fraction. Consequently, the presence of longer T_22_ and shorter relaxation times (T_2b1_, T_2b2,_ T_21_) suggests that the binding properties are stronger between water molecules and muscle tissue. The cooked sausage samples, with or without RF, GRF, or TS, showed similar trends in T2 relaxation times (Figure 1 and Figure 2A). RF and GRF did not induce significant changes in the T_2b2_ components compared to the TS samples in this study (*p* > 0.05). However, T_2b1_ was relatively lower in GRF samples compared to control, RF, and TS samples. Zheng et al. [12] also found lower T_2b1_ for chicken batters after cooking. Incorporation of GRF significantly affected the T_21_ of sausage samples compared to other samples (*p* < 0.05). The four proton populations detected in the emulsion-type sausages formulated with or without plant-based starch were designated as P_2b1_, P_2b2_, P_21_, and P_22_, as shown in Figure 2B. T2 relaxometry proportion measurements showed that the sausage samples with the highest P_21_ had the lowest P_22_ proportion. In general, T2 relaxation times demonstrated the possibility of water protons to interact with the surrounding surfaces of RF, GRF, or TS, which consequently caused some alterations in the structural space of the emulsion-type cooked sausage.

### 3.5. Protein Secondary Structures

The protein secondary structures of an emulsion-type sausage formulated with plant-based starch ingredients are shown in Table 4. Incorporation of RF or GRF in the sausage samples did not significantly influence the contents of the α-helix and β-sheet structures (*p* > 0.05). Similarly, TS did not alter the protein secondary structure (α-helix and β-sheet) of the sausage samples (*p* > 0.05) except for the β-turn and random coil structure. The similar effects observed among these ingredients could be associated with the low content of functional protein in the ingredients added to the product. However, the denaturation and aggregation process of meat protein, followed by the formation of the gel structure, suggests that the heating process is crucial to induce the gelation of protein. The secondary structure results showed that β-turn contents were lowest in emulsion-type sausages containing TS compared to RF, GRF, and the control (*p* < 0.05). The differences in the structural properties could be caused by molecular network rearrangement stimulated by the addition of different plant-based starch ingredients.

Herrero [14] reported that the proportions of β-turn structures were associated with the increase or decrease in the textural properties in the meat systems. Other authors [24] found an increase in random coil structures with the addition of hydrocolloids. This fact supports our findings, which showed that random coil structures were higher for samples treated with TS. The β-sheet formation and unfolding of the α-helix structure significantly favored the gelation of porcine myosin [25]. The improvement in the protein secondary structure of the emulsified sausage prepared with plant-based starch was not significantly promoted due to the low concentration of protein in these ingredients that could better interact with muscle protein.

### 3.6. Microstructure Evaluation

**Figure 3:**
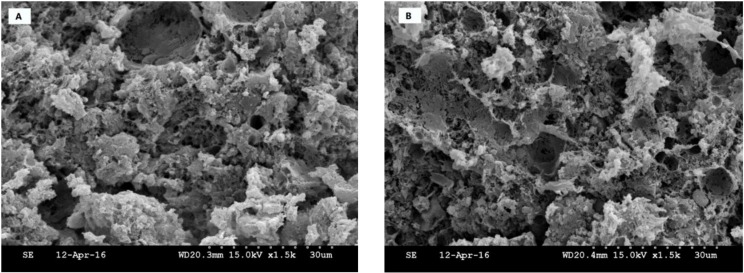

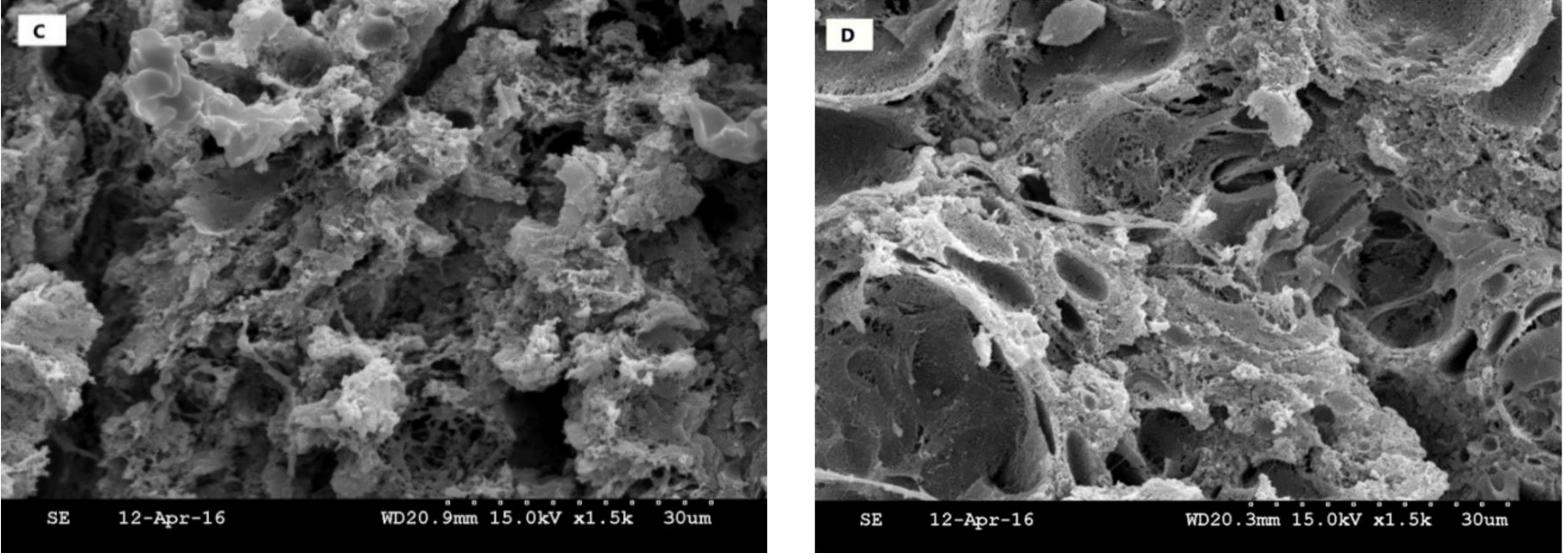
Microstructure of emulsion-type cooked sausage formulated with plant-based starch ingredients.: (**A**) control, (**B**) rice flour, (**C**) glutinous rice flour, and (**D**) tapioca starch.

Microstructural properties of emulsion-type cooked sausages formulated with RF or GRF in comparison with TS and the control, without flour or starch, are shown in Figure 3. Control samples exhibited less compact and more porous structures with several fat globules distributed in the protein network structure. Likewise, sausages formulated with RF presented various discontinuities and gaps within the structures. RF had less functionality in making a cohesive meat batter structure and a lower ability to retain water/fat when interacting with meat protein during the heating process compared to GRF and TS. Barbut et al. [16] revealed that cooked meat batter with starch and canola oil had many open channels and gaps within the structure, which affected their texture properties. TS could probably induce a more uniform and viscoelastic protein matrix compared to RF and GRF. However, it is complicated to correlate a precise modification to a specific effect of these ingredients in the emulsified meat system in the current study.

TS physically induced a greater entrapment of fat globules and promoted better interaction between muscle proteins and added components compared with RF and GRF treatments, which was reflected in lower fat loss and higher moisture retention, as observed in Table 2. Barbut et al. [16] reported that the entrapment of water/fat occurred due to the increase in the viscosity of the starch molecules in the emulsified meat product rather than starch/fat binding. As previously mentioned, the rupture and swelling of starch granules during the heating process enhances the protein matrix interaction, which generates products with better structure [4,26,27]. This process consequently reduces cooking loss and improves the microstructure of emulsified meat products. Most likely, the hydrophilic components present in starch may improve the water-holding capability of the final product by augmenting the surface area of the protein and water absorption in the systems [28]. However, the interactions between polysaccharides and meat protein upon the cooking process could be limited by diverse factors, such as functional properties, molecular size and conformation, amount of stroma and sarcoplasmic, and the meat quality, which affect the binding and viscoelastic properties of the final products.

## 4. Conclusions

The addition of RF and GRF polysaccharides had a variable impact on the quality, and functional properties of emulsion-type cooked sausage compared to TS and the control. RF and GRF successfully reduced the total fluid release and improved the composition and nutritional values of the final product. RF and GRF generated softer sausage samples compared to TS. The NMR T2 relaxation time showed that TS had more pronounced immobilized water (T_21_ component) and improved the gel network of the emulsion-type sausage in this study. RF and GRF did not induce a pronounced change in the protein secondary structure (α-helix, β-sheet, β-turn) of the emulsion-type cooked sausage. Based on our results, although TS had more functional effects on the emulsion-type sausage, it is reasonable to affirm that RF and GRF can be effectively applied as functional ingredients to improve the quality and nutritional value of emulsified meat products. However, further research is required to reinforce the functional properties of RF and GRF and to investigate the mechanism of these potential cereal flour ingredients to enhance the technological and organoleptic quality of different emulsified meat products during storage.

## Figures and Tables

**Table 1:** Formulation of emulsion-type sausage containing rice flour, glutinous rice flour, or tapioca starch.

	Lean Meat (g)	Fat (g)	Ice/Water (g)	Salt (g)	Sucrose (g)	Sodium Tripolyphosphate (g)	Pepper (g)	Flour or Starch (g)
Control	800	200	200	15	2.5	3	2.5	-
RF	800	170	200	15	2.5	3	2.5	30
GRF	800	170	200	15	2.5	3	2.5	30
TS	800	170	200	15	2.5	3	2.5	30

Control—Sausage formulated without flour or starch; RF—rice flour; GRF—glutinous rice flour; TS—tapioca starch.

**Table 2:** Nutritional composition and characteristics of emulsion-type sausages formulated with rice flour, glutinous rice flour, or tapioca starch.

	Control	RF	GRF	TS
Nutritional composition				
pH	6.57 ± 0.01 ^c^	6.63 ± 0.02 ^b^	6.68 ± 0.01 ^a^	6.69 ± 0.01 ^a^
Protein (%)	15.55 ± 0.23 ^a^	15.04 ± 0.18 ^b^	15.59 ± 0.32 ^a^	14.75 ± 0.10 ^b^
Moisture (%)	64.35 ± 0.08 ^b^	65.72 ± 0.49 ^a^	65.17 ± 0.52 ^a^	65.36 ± 0.54 ^a^
Fat (%)	18.70 ± 0.04 ^a^	16.04 ± 0.64 ^b^	16.37 ± 0.60 ^b^	16.43 ± 0.65 ^b^
Ash (%)	1.92 ± 0.17 ^a^	2.08 ± 0.30 ^a^	1.96 ± 0.08 ^a^	1.92 ± 0.17 ^a^
Cooking quality (%)				
Cooking Yield	83.75 ± 0.44 ^d^	88.04 ± 0.49 ^c^	94.29 ± 0.33 ^b^	98.62 ± 0.08 ^a^
Moisture retention	53.90 ± 0.28 ^d^	58.13 ± 0.26 ^c^	61.45 ± 0.21 ^b^	64.46 ± 0.05 ^a^
Emulsion Stability (%)				
TFR	9.72 ± 0.51 ^a^	7.87 ± 1.01 ^b^	3.15 ± 0.42 ^c^	1.47 ± 0.58 ^d^
Fat loss	1.10 ± 0.04 ^a^	0.74 ± 0.07 ^b^	0.30 ± 0.02 ^c^	0.37 ± 0.07 ^c^

Control—sausage formulated without flour or starch; RF—rice flour; GRF—glutinous rice flour; TS—tapioca starch; and TFR—total fluid release. ^a–d^: Means ± SD values of different superscripts within the same row are significantly different (*p* < 0.05).

**Table 3:** Textural properties of emulsion-type cooked sausages formulated with rice flour, glutinous rice flour, or tapioca starch.

	Control	RF	GRF	TS
Hardness (N)	46.14 ± 4.30 ^a^	50.41 ± 213.84 ^b^	44.95 ± 2.96 ^a^	65.92 ± 3.73 ^c^
Springiness (mm)	0.84 ± 0.03 ^a^	0.82 ± 0.04 ^a^	0.81 ± 0.03 ^a^	0.91 ± 0.01 ^b^
Cohesiveness	0.52 ± 0.04 ^a^	0.51 ± 0.07 ^a^	0.52 ± 0.06 ^a^	0.69 ± 0.05 ^b^
Chewiness (N)	19.96 ± 2.71 ^a^	21.13 ± 3.01 ^a^	18.84 ± 2.58 ^a^	41.58 ± 4.76 ^b^

Control—sausage formulated without flour or starch; RF—rice flour; GRF—glutinous rice flour; TS—tapioca starch; ^a–c^: Means ± SD values of different superscripts within the same row are significantly different (*p* < 0.05).

**Table 4:** Relative percentages of protein secondary structures of an emulsion-type sausage formulated with rice flour, glutinous rice flour, or tapioca starch.

	α-Helix (%)	β-Sheet (%)	β-Turn (%)	Unordered (%)
CONTROL	40.50 ± 9.88 ^a^	25.91 ± 8.15 ^a^	18.38 ± 4.42 ^a^	10.77 ± 0.47 ^b^
RF	44.87 ± 6.92 ^a^	22.17 ± 5.02 ^a^	15.45 ± 1.57 ^a^	12.15 ± 1.79 ^b^
GRF	48.12 ± 1.66 ^a^	25.42 ± 2.47^a^	12.16 ± 3.23 ^a^	11.13 ± 0.78 ^b^
TS	43.20 ± 6.82 ^a^	28.53 ± 10.69 ^a^	10.37 ± 5.67 ^b^	15.08 ± 6.30 ^a^

Control—samples formulated without flour or starch; RF—rice flour; GRF—glutinous rice flour; TS—tapioca starch. ^a–b^: Means ± SD values with different superscripts within the same column are significantly different (*p* < 0.05).
